# Isolation and characterization of apical papilla cells from root end of human third molar and their differentiation into cementoblast cells: an in vitro study

**DOI:** 10.1186/s12575-023-00190-6

**Published:** 2023-01-23

**Authors:** Morvarid Ebadi, Amirfarhang Miresmaeili, Sarah Rajabi, Shahrokh Shojaei, Sareh Farhadi

**Affiliations:** 1 Department of Biomedical Engineering, Central Tehran Branch, Islamic Azad University, Tehran, Iran; 2 Orthodontic Department of Hamadan University of Medical Sciences and Hamadan Dental Research Centre, Hamadan, Iran; 3 Department of Cell Engineering, Cell Science Research Center, Royan Institute for Stem Cell Biology and Technology, ACECR, Tehran, Iran; 4 Stem Cells Research Center, Tissue Engineering and Regenerative Medicine Institute, Central Tehran Branch, Islamic Azad University, Tehran, Iran; 5 Department of Oral & Maxillofacial Pathology, Faculty of Dentistry, Tehran Medical Sciences, Islamic Azad University, Tehran, Iran

**Keywords:** Stem cells, Apical papilla, Cementoblasts, Differentiation

## Abstract

**Background:**

Periodontal regeneration, treatment of periodontal-related diseases and improving the function of implants are global therapeutic challenges. The differentiation of human stem cells from apical papilla into cementoblasts may provide a strategy for periodontitis treatment. This study aimed to evaluate the differentiation of primary human stem cells apical papilla (hSCAPs) to cementoblast cells.

**Material and methods:**

SCAPs cells were isolated from human third molar and then incubated for 21 days in a differentiation microenvironment. Alkaline phosphatase (ALP) and Alizarin red S staining assays were performed to evaluate the calcium deposition and formation of hydroxyapatite in the cultured hSCAPs microenvironment. Real-time polymerase chain reaction (RT-PCR) assay was performed for cementum protein 1 (CEMP1), collagen type I (COL1), F-Spondin (SPON1), osteocalcin (OCN), and osteopontin (OPN) as specific markers of cementoblasts and their progenitors.

**Results:**

ALP phosphatase activity in day 21 of treatment demonstrated a significant increase in ALP compared to the control. Alizarin red S staining assay showed that the differentiated hSCAPs offered a great amount of calcium deposition nodules compared to the control. The increased expression level of CEMP1, OCN, OPN, COL1 and Spon1 was observed in days 7, 14 and 21 compared to the control, while greatest expression level was observed in day 21.

**Conclusion:**

In conclusion, the differentiation microenviroment is convenient and useful for promoting the differentiation of hSCAPs into cementoblast**.**

## Highlights

• Human stem cells from apical papilla cells were isolated from a human third molar

• Human stem cells from apical papilla were successfully differentiated into cementoblast cells

## Introduction

Periodontitis is one of the most common infectious diseases characterized by chronic inflammation and the destruction of supporting periodontal tissues [1–3]. Gram-negative bacteria mainly cause periodontitis in the subgingival biofilm [1, 4]. Severe periodontitis affects approximately 11% of the world's population and is the sixth most common human disease [5]. Age, smoking, and numerous systemic disorders such as diabetes, rheumatoid arthritis, neurodegenerative disorders, cardiovascular diseases, and pregnancy are risk factors for periodontitis [6, 7]. This disease, causes irreversible damage to the tooth's supporting tissues, including the periodontal ligament (PDL), alveolar bone, and cementum (cementoblasts), which can lead to premature tooth loss [8]. Mechanical removal of bacterial biofilms by scaling and root planning [9], bone grafting, guided tissue/bone regeneration (GTR/GBR), enamel matrix derivative (EMD), and platelet-rich plasma are regenerative treatments for periodontitis (PRP). Despite regenerative therapies, restoring the periodontium completely and consistently is impossible, particularly in cases where the illness has produced significant periodontal tissue abnormalities[4, 10]. Therefore, dental implants are considered the most appropriate treatment option for tooth loss. Overall, the high success rates, predictability of the procedure, and low incidence of complications during and after dental implant placement have made this procedure accessible and effective for the general population. Despite the high success rate of dental implants and their widespread use, there are several problems including the quality and quantity of bone in the recipient site and the lack of bone integrity [11]. In addition, unlike natural teeth, implants do not have important functional components such as cementum and PDL and are therefore unable to dampen chewing force [1]. At this time, various studies have focused on the using of different materials with specific surface properties to improve bone integrity [11].

The surface quality of implants plays a key role in their long-term clinical success [12]. Surface modification of dental implants is performed to improve or accelerate osseointegration, enhance biocompatibility, and prevent the bacterial adhesion which significantly affect their clinical applications. Various methods such as sandblasting, acid etching, plasma etching, plasma spray deposition, sputtering deposition, and cathodic arch deposition have been employed to improve the performance of dental implants [13]. One of the main factors in the success of dental implant insertion is osseointegration. The surface properties of a dental implant and its macroscopic structure are the important the osseointegration process [14]. Therefore, considering the role of cementum in the attachment of periodontal ligament to the alveolar bone and tooth root, the modification of implant surface with cementoblasts can improve and accelerate the osseointegration [15, 16].

However, due to the inability to isolate and cultivate the cells required to produce cementum, the differentiation of stem cells into cementoblasts seems to be important [15, 17]. Cementoblasts can be isolated from molar and premolar periodontal ligaments [15, 16], Dental-derived mesenchymal stem cells (DMSCs), including mesenchymal stem cells isolated from dental pulp, periodontal ligament, tooth follicle, apical papilla, and gingiva have multiple differentiation capabilities [18]. Among them, stem cells are generated from the apical papilla (SCAPs) as a source of DMSCs of immature permanent teeth before the teeth erupt into the oral cavity [19]. These cells have different characteristics such as great proliferative potential, self-renewal ability, and low immunogenicity [20]. SCAPs can differentiate into various lineages of cells in vitro including osteoblasts, odontoblasts, neural cells, adipocytes, chondrocytes, and hepatocytes, and they can modulate root development [21–23].

Kitagawa et al*.* extracted cementoblast cells from premolar teeth and showed that the human cementoblast-like cell line could be utilized effectively for the proliferation and differentiation of human cementoblasts [17]. Moreover, Carvalho et al*.* demonstrated that the differentiation of rat molar periodontal ligament cells to cementoblast cells increased the potential for cementum regeneration [15]. Some studies have utilized the differentiation of human cells into cementoblasts to enhance the applications of dental implants.

In this study, apical papilla stem cells were differentiated to cementoblasts using a differentiation microenvironment containing ascorbic acid and ß–glycerophosphate. The differentiation of SCAPs was confirmed by using the Alizarin red S staining assay. The alkaline phosphatase (ALP) was employed to determine the degree of mineralization and calcium hydroxyapatite formation. Real-time PCR was conducted to evaluate the expression of COL1 (collagen type I), F-Spondin (SPON1), osteocalcin (OCN), osteopontin (OPN), and cementum protein 1 (CEMP1).

## Materials and methods

Dulbecco's Modified Eagles Medium (DMEM), penicillin/streptomycin (5,000 units penicillin and 5 mg streptomycin/mL, USA), fetal bovine serum (FBS), and Trypsin–EDTA (0.25%) were obtained from Gibco (Invitrogen, USA). Dimethyl Sulfoxide (DMSO), Ascorbic acid, and ß–glycerophosphate were purchased from Sigma Aldrich (USA). Alizarin red S was provided by Merck Co. (Germany). Cell lysis buffer was obtained from Abcam Co. (USA). An alkaline phosphatase assay kit was obtained from Pars Azmoon Co. (Iran). DEPC-treated water was provided by Thermo Fisher Scientific Co. (USA). Qiazol and cDNA synthesis kits were obtained from Kiazist Co. (Iran) and Parstous Co. (Iran), respectively. Chloroform, isopropanol, and etanol absolute alcohol were provided from Merck Co. (Germany). SYBR Master with high Rox (2X) was obtained from Addbio Co. (Korea). The primers of COL1, SPON1, OCN, OPN, and CEMP1 genes were prepared by Sinaclon Co. (Iran).

### Extraction and SCAPs culture

All research protocol of this study were approved by the Ethics Committee of Islamic Azad University, Central Tehran Branch (Co.IR.IAU.CTB.REC.1400.110). The tooth roots of molar teeth which extracted due to orthodontic treatment were harvested for stem cells. The apical part of the extracted tooth was rinsed with phosphate-buffered saline (PBS) and then stored and incubated in a digestion solution containing 1 mL of PBS and 3 mg/mL of type II collagen at 37 °C for 1 h. After centrifuging, the resulting solution was transferred into a T25 flask containing DMEM-F12 medium. The resulting samples were cultured at 37 °C and 5% CO_2_ in an incubator [15]**.**

When the confluency of the cells reached 90%, the samples were washed with PBS. Then, the solution was added to a mixture of Trypsin/EDTA (0.25%). The solution was finally incubated for 5 min at 37 °C. After separating the cells from the bottom of flask, they were transferred to a 15 mL Falcon containing DMEM solution with 10% FBS and 1% penicillin/streptomycin. The samples were centrifuged for 10 min at 1800 rpm. The cells (1 × 10^5^ cells/cm^2^) were cultured in a T25 flask till it reaches to 3 × 10^5^ cells/cm^2^ and then transferred to the T75 flask [24]. The cells were passaged in DMEM-Low glucose medium containing 10% FBS and 1% penicillin/streptomycin. Passage 4 was used to induce differentiation of the cells [25].

Flow cytometry (BD FACS Calibur instrument, BD, biosciences San Jose, CA) was used to identify CD90, CD73, and CD34 as stem cell and hematopoietic cell markers, respectively, in order to confirm the stem cell nature of the examined cells. Therefore, cells were cultured to tyrosinase passage 3, and after centrifuging at 1800 rpm for 5 min, the supernatant was discarded, and the precipitate was dissolved in PBS and centrifuged once more. Next, the cell sediment was dissolved in 1 μL, and FlowJo™ v10 Software was used to analyze the data (7.0, Team FlowJo at CYTO 2019, Vancouver, BC) [26].

### Preparation of differentiation microenvironment

After 4-Th passages, cultured stem cells (1 × 10^5^ cells/cm^2^) were transferred into a 6-wells plate and then cultured in DMEM-Low glucose medium containing 10% FBS and 1% pen strap [27]. After 48 h which the cell density reached 80%, the culture microenvironment was replaced with a differentiation microenvironment containing DMEM with 10% FBS, penicillin/streptomycin 1%, 10 mM of ß-glycerophosphate, and 50 g /mL of ascorbic acid 2-phosphate (Sigma-Aldrich Co. Louis, MO, USA). Till 21-St. day, the microenvironment culture was replaced every three days [28].

### Alizarin red S staining assay

In order to evaluate the extracellular calcium deposition and formation of hydroxyapatite calcium crystals, Alizarin red S staining was performed according to the method described by Asl et al., with minor modifications [29]. After 21 days of cell differentiation, the Alizarin red S staining assay identified the calcium deposits. After cell differentiation, the microenvironment that promoted differentiation was eliminated. The cells were washed with PBS, fixed for 15 min in the dark condition with 10% formaldehyde, and washed twice with distilled water. The calcified nodules were stained for 30 min at pH 4.1 using 1% Alizarin red S dye dissolved in water. The stained cells were then washed twice with distilled water and examined under a labomed-model light microscope with a magnification of 20 μm. Afterward, for quantitative analysis of calcium mineralization, 200 μL acetic acid (10%, v/v) was added to each well, samples were incubated for 1 h at room temperature, and cell layers were then separated from the bottom of the plate. They were poured into a microtubule and centrifuged for 15 min at 15 °C and 1500 rpm. Finally, the adsorption of the resulting solution was performed at 570 nm using an Elisa reader.

### Alkaline phosphatase assay

The alkaline phosphatase (ALP) assay was performed according to the method described by Asl et al., to evaluate the differentiation of apical stem cells into hard tissue [29]. After 21 days, ALP activity was measured using an alkaline phosphatase kit (Pars Azmoun, Iran) according to the manufacturer's instructions. At day 21, the differential microenvironment was removed and the cells were washed with PBS. The cells were lysed with Trypsin–EDTA (0.25%) and lysate buffer 60 (µL) and Bradford assay was also performed to evaluate their protein content [30]. In order to investigate the alkaline phosphatase activity, 25 g of protein from each sample along with alkaline phosphatase kit solutions were read-out at 405 nm using the Elisa reader.

### Quantitative real-time polymerase chain reaction (qRT-PCR) assay

Total RNA was extracted from differentiated cementoblast cells in days 7, 14, and 21 using a Qiazol kit (Kiazist, IRAN, Tehran). The purity and concentration of the extracted RNA samples were evaluated using NanoDrop (Thermo Fisher Scientific, Waltham, MA) device. Extracted RNA (1 µg) was utilized to synthesize cDNA according to the kit's instructions (Parstous, cDNA, Iran, Synthesis Kit). The expression of SPON1, CEMP1, GAPDH, OPN, OCN, and COL1 for human cementoblast cells was evaluated using Sinacolon kit according to the manufacturer's instruction (Sinaclon, Iran). The expression level of primers was normalized by glyceraldehyde 3-phosphate dehydrogenase control primer (GAPDH) [31]. Table 1 indicates the sequence of the studied primers. A mixture of PCR components, including SYBR Master with high Rox (addbio, Korea), dehydrated water, and primers, was formulated for each gene. The following temperature programming was used during the analysis:

**Table1:** Cementoblast cells primer sequences utilized in the RT-PCR

Reverse sequence	Forward sequence	RT-PCR primer set
5'TCTGGGGTGATTGTCTTGACTT3'	5' GACCATCCTATCTCTTTGGACCT 3'	CEMP1
5'ACATTGCTAAAGTGGGTGCTTC3'	5' GCTTGTTCTAGGAGGGACCATT3'	SPON1
5' AGGAGAGCCATCAGCACCT3'	5' ATGCCTGGTGAACGTGGT3'	COL1
5'TCAGCCAACTCGTCACAGTC 3'	5' GCCGAGGTGATAGTGTGGTT 3’	OCN
5' TGAGGTGATGTCCTCGTCTG3’	5' GCCGAGGTGATAGTGTGGTT 3’	OPN
5'CTTCCTCTTGTGCTCTTGCT3'	5'CTCATTTCCTGGTATGACAACGA3'	GAPDH

Initial denaturation occurred at 95 °C for 5 min, followed by 40 cycles of amplification at 95 °C for 15 s, 60 °C for 20 s, and 72 °C for 30 s, as well as a melting curve from 65 °C to 95 °C. The obtained data were registered, including CT numbers (threshold cycle), proliferation, and melting curves for each gene. The 2^−ΔΔCT^ equation was used to analyze the CT numbers.

### Statistical analysis

All data were analyzed using SPSS software (version 22). Using one-way ANOVA, the data were reported as mean ± standard deviation (SD). Statistical differences between the data were analyzed. The level of statistical significance was expressed as *P* ≥ 0.05.

## Results

### Extraction and culture of SCAPs

**Fig. 1:**
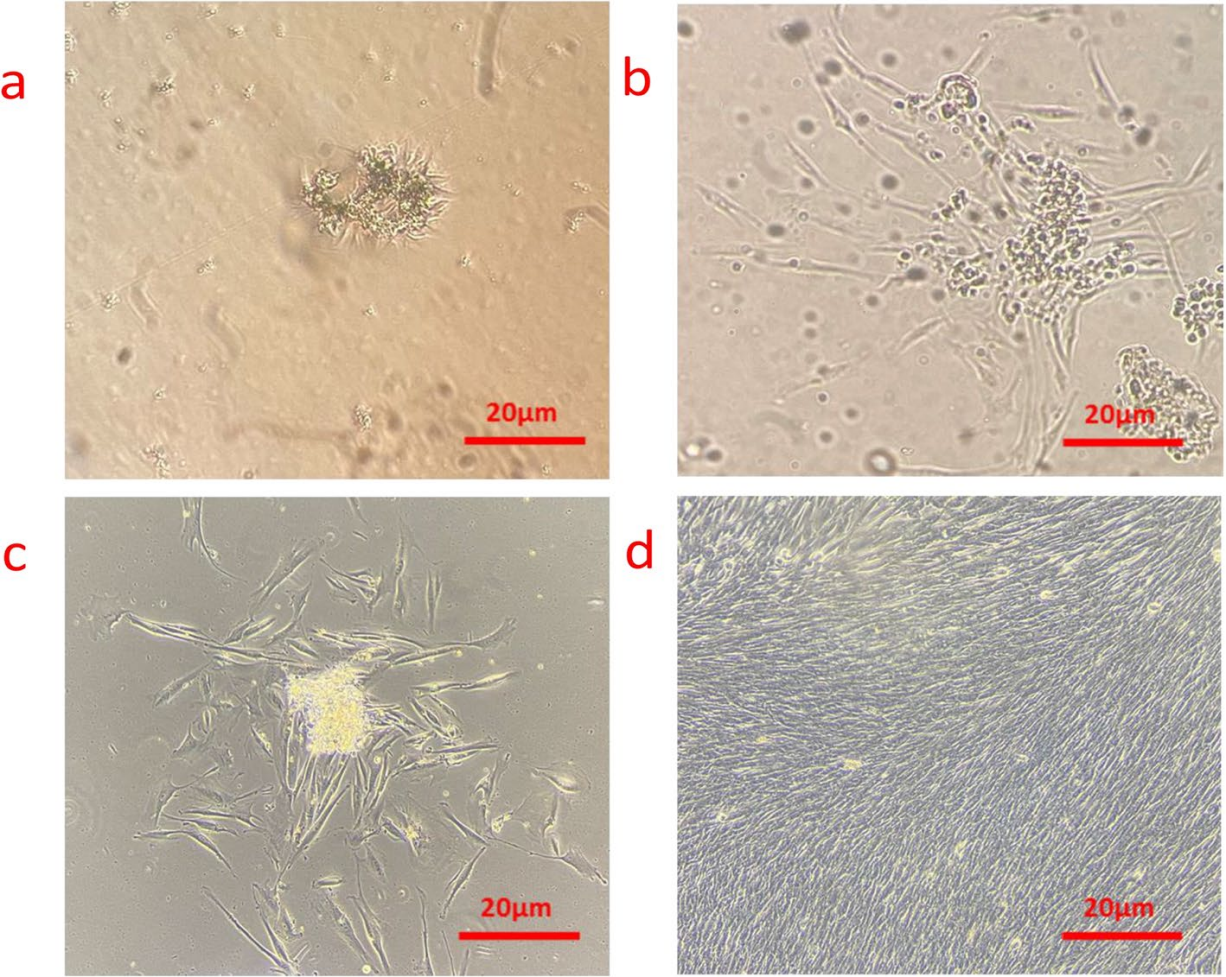
Light microscopy image of extracted SCAPs cells from human apical teeth of 3rd molar root after: **a** 2 days of extraction in DMEM solution with 10% FBS and 1% penicillin/streptomycin.; **b** 72 h of cell culture in DMEM solution with 10% FBS and 1% penicillin/streptomycin.; **c** 4 days of cell culture in DMEM solution with 10% FBS and 1% penicillin/streptomycin.; **d** 5 days of cell culture in DMEM solution with 10% FBS and 1% penicillin/streptomycin

In the present study, cells were extracted and cultured from the apical part of human third molars. The initial phases of stem cell culture in a T25 flask are depicted in Fig. 1a-d.

### Flow cytometry

**Fig. 2:**
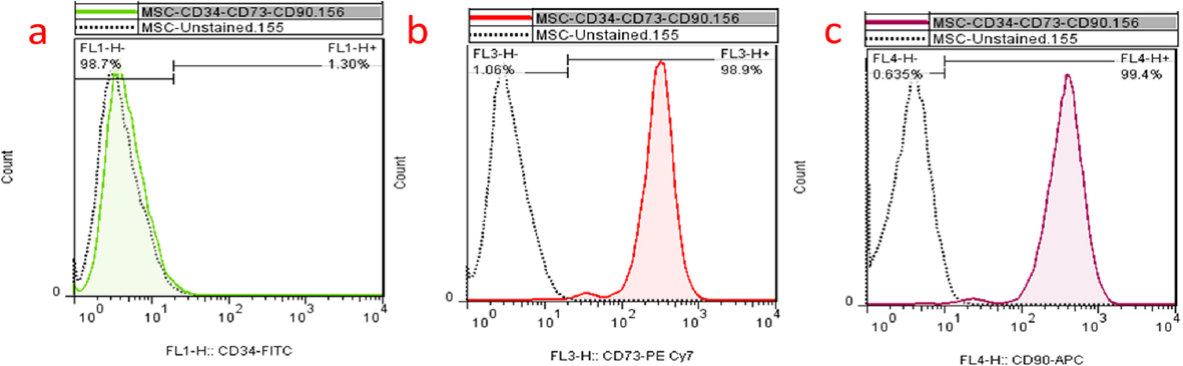
Flow cytometry analysis of extracted SCAPs cells from human apical teeth of 3rd molar root after: **a** CD34.; **b** CD90 and **c** CD73, which CD90 and CD73 are positive markers, and CD34 marker is negative marker. SCAPs cells expressed CD73 and CD90, which are associate with MSC phenotype

Flow cytometry analysis revealed that 99.4%, 98.9%, and 1.30% of stem cells isolated from the apical portion of human third molars in passage 3 expressed the markers CD90, CD73, and CD34, respectively. At the tooth roots of human third molars, CD90 and CD73 markers were positive for stem cells, while CD34 marker was negative. The results of this study are shown in Fig. 2.

### Alizarin red S staining assay

**Fig. 3:**
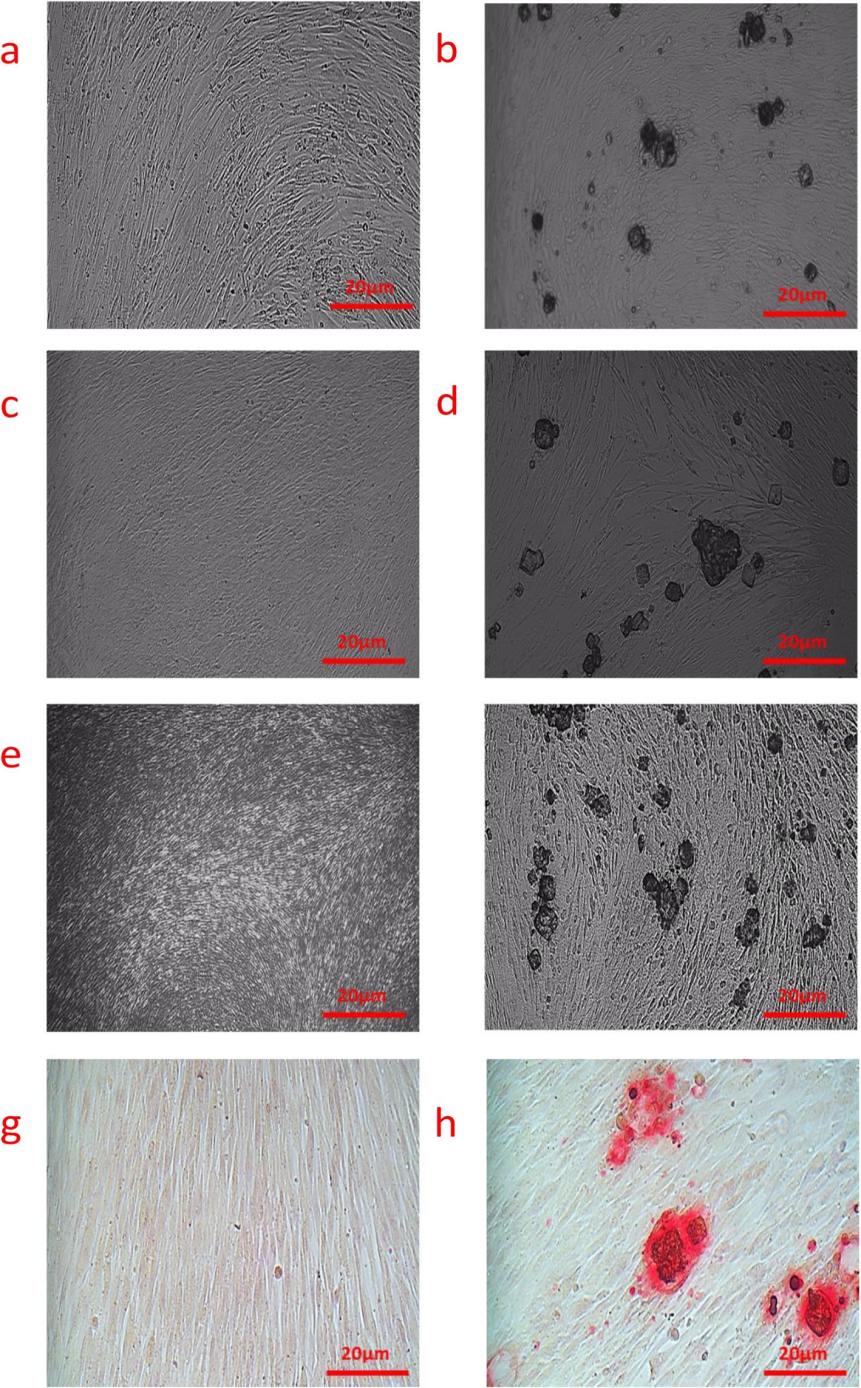
Light microscopy images of differentiation SCAPs cells to cementoblast, with Alizarin red staining assay. **a** Control cells on day 7 of cell culture in DMEM solution with 10% FBS and 1% penicillin/streptomycin.; **b** Differentiated cells on day 7 of cell culture in DMEM with 10% FBS, penicillin/streptomycin 1%, 10 mM of ß-glycerophosphate, and 50 g/mL of ascorbic acid 2-phosphate.; **c** Control cells on day 14 of cell culture in DMEM solution with 10% FBS and 1% penicillin/streptomycin.; **d** Differentiated cells on day 14 of cell culture in DMEM with 10% FBS, penicillin/streptomycin 1%, 10 mM of ß-glycerophosphate, and 50 g/mL of ascorbic acid 2-phosphate.; **e** Control group on day 21 of cell culture in DMEM solution with 10% FBS and 1% penicillin/streptomycin.; **f** Differentiated cells on day 21 in DMEM with 10% FBS, penicillin/streptomycin 1%, 10 mM of ß-glycerophosphate, and 50 g/mL of ascorbic acid 2-phosphate.; **g** Stained control cells on day 21, and (**h**) Stained differentiation SCAPs cells on day of 21

The control cells in days 7, 14, and 21 are depicted in Fig. 3 a, c and e, respectively. Figure 3b, d, and f depict the differentiated cells in days 7, 14, and 21. As shown in Fig. 3b, hydroxyapatite crystals initiate to form in day 7 and were clearly visible in day 21.

The control and differentiation samples stained with Alizarin red S after 21 days are depicted in Figs. 3g and h, respectively. As shown in Fig. 3h, the stained masses are calcium hydroxyapatite crystals. Differentiation cells were counted using Image J software, and the results indicated that 48.18 -51.37% of the cells were differentiated.

**Fig. 4:**
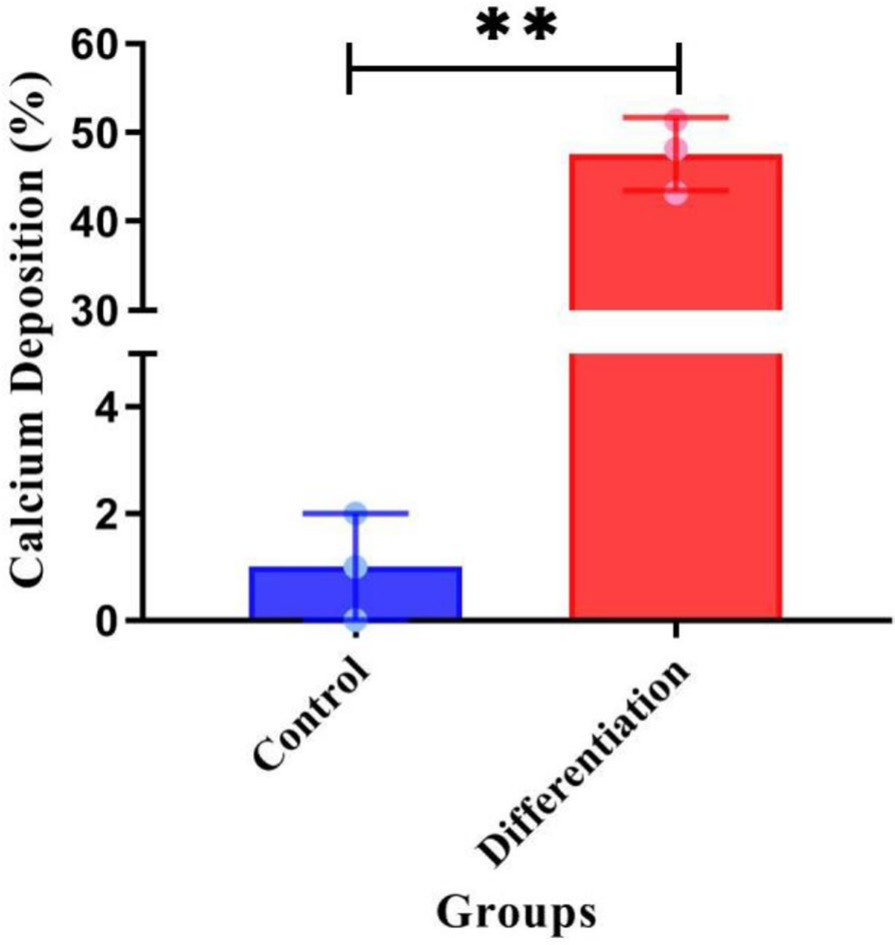
Alizarin red S test of control and differentiated cells after 7, 14 and 21 days of differentiation. In 21-St. day of differentiation, the Alizarin red S staining test identified calcium deposition. **a** control (1.00 ± 0.81); **b** differentiated cells (47.59 ± 3.35) *n* = 3, (*p* < 0.001). The control and differentiation samples stained with Alizarin red S after 21 days are depicted

Figure 4 shows the results of the Alizarin red S assay in the differentiated cell group compared to the control. The results of this study revealed a statistically significant difference between the differentiated and control groups, with calcium deposition in the differentiated group as significantly greater than the control group (*p* < 0.001). The differentiated hSCAPs clearly revealed a great amount of calcium deposition nodules (47.5%) compared to the control (1.0%).

### Alkaline phosphatase assay

**Fig. 5:**
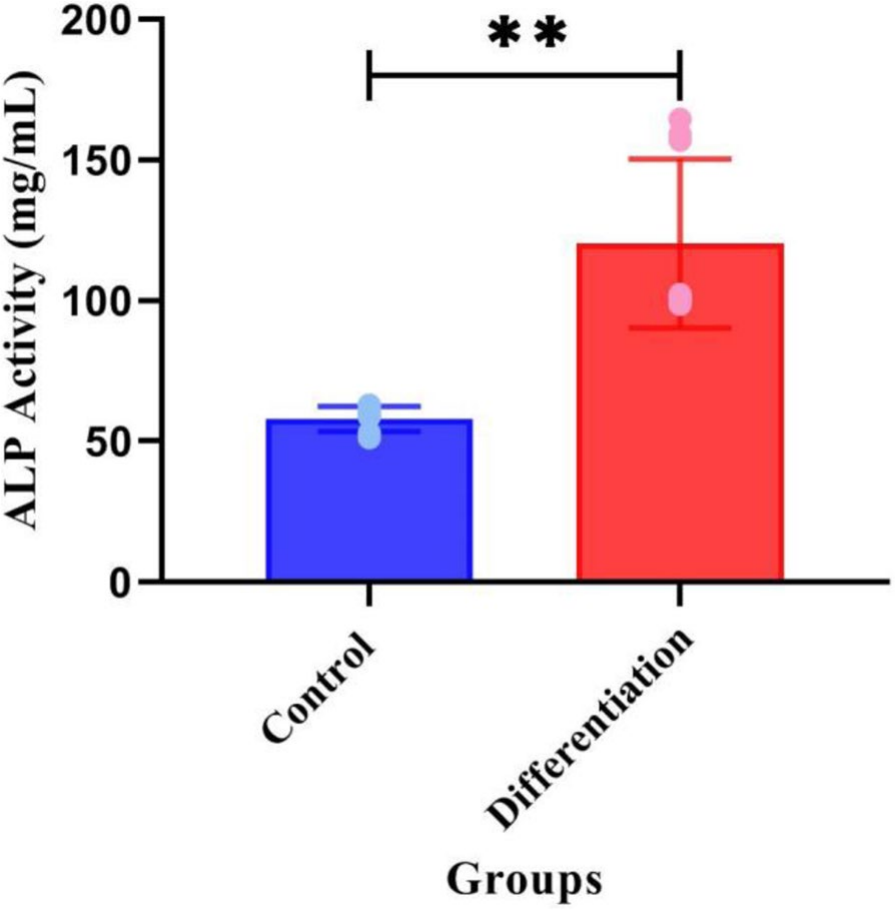
Differentiation of cementoblasts on day 21. Alkaline phosphatase activity assay for different group compared to the control group by one-way ANOVA test. ALP control: 1.01 ± 0.07, differentiation: 1.99 ± 0.47, *n* = 3, (*p* < 0.001)

The present study demonstrated that ALP activity increased in day 21. As shown in Fig. 5, the differentiation group had the greatest level of mineralization compared to the control (*p* < 0.001). More alkaline phosphatase was produced by differentiated cells (2.05%) than by control cells (1.01%).

### Quantitative real-time polymerase chain reaction (qRT-PCR) assay

**Fig. 6:**
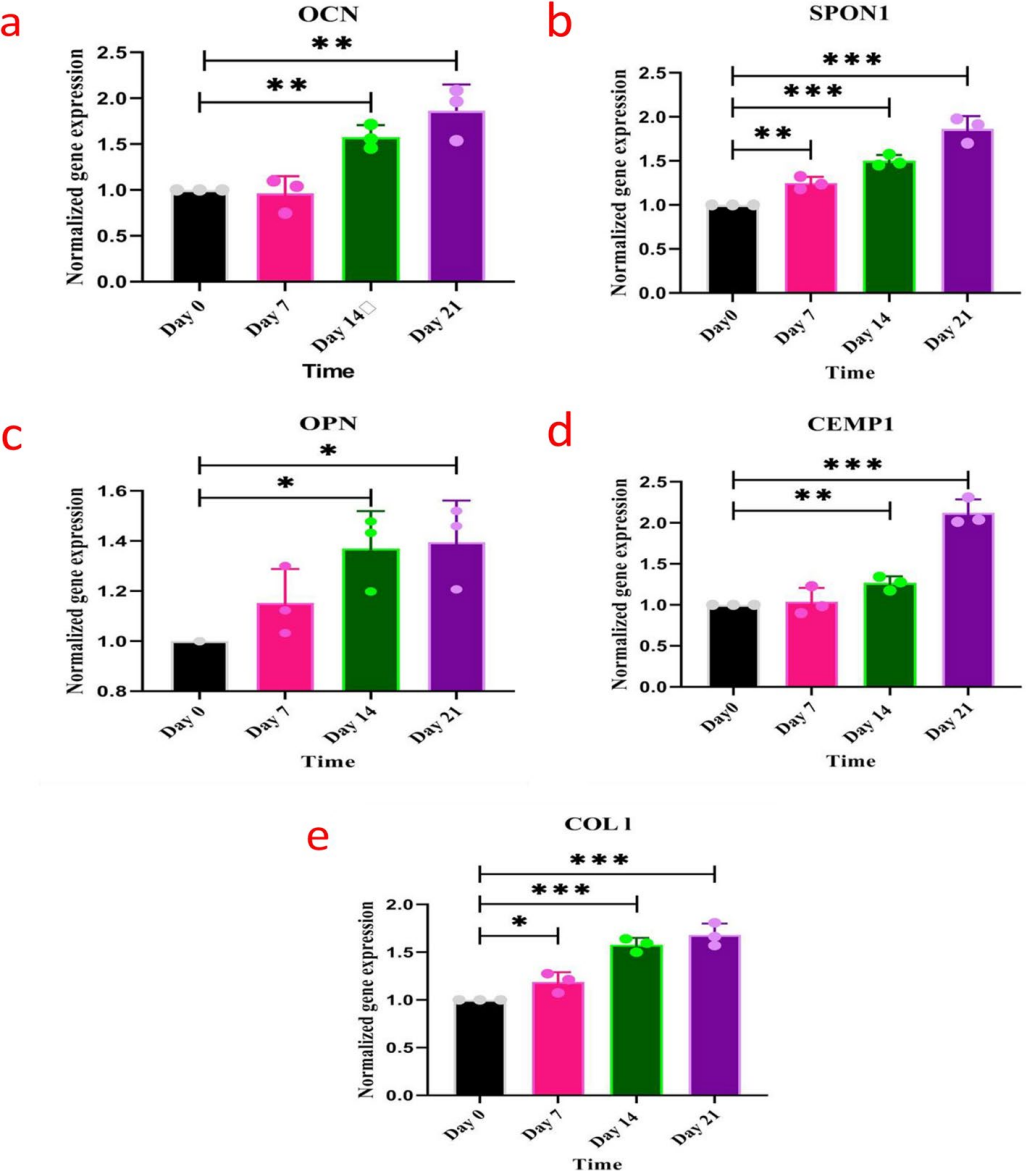
Assessment of cementoblast cells using RT-PCR test on 0, 7, 14 and 21 days for: **a** OCN (*p* < 0.007); **b** SPON1 (*p* < 0.0005).; **c** OPN (*p* < 0.01).; **d** CEMP1 (*p* < 0.0003) and **e** COL1 (*p* < 0.0006), in different groups compared with the control group by one-way ANOVA test (0 day indicates controls)

GAPDH was used as the reference gene in a qRT-PCR reaction along with OPN, OCN, F-Spondon (SPON1), CEMP1, and COL1 primers. As shown in Fig. 6, each primer in the extracted RNA of control and differentiation groups was evaluated in days 7, 14, and 21. The differences between the differentiated cells and controls were reported using a logarithmic scale. The expression of OCN gene was significantly greater in the differentiated samples in days 7, 14, and 21 compared to the control (*p* < 0.007). In days 7, 14, and 21, the expression level of OCN was approximately 0.96, 1.57, and 1.86 folds than control, respectively. In addition, OCN expression levels in days 14 and 21 were 1.63 and 1.93 folds greater than its level at day 7 (Fig. 6a). As shown in Fig. 6b, SPON1 was a specific primer of the cementoblast cells that were differentiated from the control (*p* < 0.0005). This increase was also observed for SPON1 gene, whose expression level in days 7, 14, and 21 was 1.24, 1.33, and 1.86 folds greater than the control, respectively. In addition, the greatest expression level of this gene was observed in days 14 and 21, which were 1.07 and 1.39 folds greater than in day 7, respectively. Regarding the OPN gene, the expression of this gene in days 7, 14, and 21 was approximately 1.15, 1.36, and 1.39 folds greater than that of the control, respectively (*p* < 0.01). The expression levels of the OPN gene in days 14 and 21 were 1.18 and 1.20 folds greater than that obtained in day 7, respectively (Fig. 6c). Moreover, CEMP1 gene expression in differentiated samples in days 7, 14, and 21 showed a significant increase compared to the control (*p* < 0.0003) (Fig. 6d). At days 7, 14, and 21, the expression of this gene was approximately 1.03, 1.26, and 2.12 folds that of the control, respectively. In addition, the expression levels of the CEMP1 gene were 1.22 and 2.05 folds greater in days 14 and 21 than in day 7. COL1 expression increased significantly in days 7, 14, and 21 compared to the control (Fig. 6e) (*p* < 0.0006). Therefore, gene expression levels in days 7, 14, and 21 were approximately 1.18, 1.64, and 1.68 folds that of the control. In addition, the level of COL1 expression in days 14 and 21 was 1.38 and 1.42 folds greater than its level in day 7.

## Discussion

The differentiation of stem cells into cementoblasts and their use in dental implants could increase their cementum regeneration potential. Due to the inability to isolate and cultivate cementoblasts, few studies have examined the formation and function of root cementum [15]. Therefore, differentiating mesenchymal stem cells into cementoblasts can be helpful in periodontal repair through regenerative therapies. Although previous studies have examined the differentiation of a wide range of stem cells, no study has examined the differentiation of stem cells from apical papillary into cementoblasts. This paper investigated the differentiation of cementoblasts in response to ß-glycerophosphate and ascorbic acid 2- phosphate in cultured hSCAPs. Alizarin red S staining, Alkaline phosphatase, and qRT-PCR assays confirmed the differentiation microenvironment successfully differentiated hSCAPs into cementoblasts. These results may be due to the role of ascorbic acid as co-factor for hydroxylation of proline and lysine in procollagen [32] and secretion of COL1 into the extracellular matrix (ECM) [33], which is involved in the differentiation of hSCAPs to osteoblasts and cementoblasts [34]. ß-glycerophosphate can play an essential role in the osteogenic differentiation of MSCs [35]. By increasing the secretion of COL1, ascorbic acid increases intracellular signaling and ultimately facilitates bone differentiation. β-Glycerophosphate works as a source of phosphate for bone mineralization and induces the bone gene expression by kinase phosphorylation, despite the fact that phosphate source is required for the production of hydroxyapatite mineral, recent findings have shown that mineral phosphate (Pi) acts as an intracellular signaling molecule to regulate the expression of osteogenic genes, including osteopontin[36].

Flow cytometry results demonstrated that apical papillary stem cells were successfully extracted from the third molar and differentiated into cementoblasts. Previous studies have examined the differentiation of various types of mesenchymal stem cells to cementoblasts, such as dental pulp stem cells (DPSCs)[36], periodontal ligament stem cells (PDLSCs)[37], dental follicle stem cells [38], bone marrow mesenchymal stem cells [39] and cranial neural crest-derived cells (CNCCs)[40]. Since the apical papilla contains more mesenchymal stem cells (MSCs) than mature dental pulp tissue, hSCAPs were utilized [20]. In addition, cryopreservation does not affect the biological and immunological properties of SCAPs; thus, they can be cryopreserved for future clinical applications while retaining their regenerative potential [41]. hSCAPs demonstrated a greater proliferation rate than DPSCs and PDLSCs [20, 21, 42, 43], and possessed a greater migration capacity than DPSCs [20, 23].

The ability of osteoblasts to produce mineralized matrix and calcium deposition is known as extracellular matrix (ECM) mineralization [44, 45], which can be identified as reddish-black spots using Alizarin red S staining [46]. Here, we used the Alizarin red S staining assay to evaluate the calcium deposition rate and formation of hydroxyapatite calcium in the cultured hSCAPs within 21 days in a differential microenvironment [29]. As shown in Fig. 4, differentiated hSCAPs contained a more significant number of calcium deposition nodules than the control, confirms the mineralization role of ß-glycerophosphate and ascorbic acid 2-phosphate in differentiated hSCAPs. Bao et al. examined the differentiation of cementoblasts with Alizarin red S staining assay, wherein the amount of hydroxyapatite in the used cementum was determined to be between 45 and 50%, which is consistent with our findings [47]. This result indicates that cementoblast cells were generated successfully.

ALP, a membrane-bound glycoproteins enzyme found in mineralized sites, is an early marker of osteoblast, odontoblast, and cementoblast differentiation [29, 31, 48, 49]. During the bone regeneration, ALP secretion plays a crucial role in the mineral deposition of matrix [29, 50]**.** Herein, using ALP activity assay, we compared alkaline phosphatase produced in differentiated samples to the control. This study revealed a significant increase between the differentiated and control group. Our findings demonstrated that ß-glycerophosphate and ascorbic acid 2-phosphate play a crucial role in inducing cementoblast differentiation. As argued in [48, 51–53], the secretion of alkaline phosphatase can be evaluated from one week of differentiation process and increases and reaches its greatest level at 14 and 21 days after differentiation progression. Therefore, in our study, 21-St. day have been chosen for ALP evaluation. Kadokura et al. demonstrated that the number of positive ALP cells in human periodontal ligament cells cultured for 21 days in a differential microenvironment increased in the 21-St. day, which is consistent with our findings [48].

Based on the previous studies, cementum protein 1 (CEMP1), cementum attachment protein (CAP), bone-related genes (Alkaline phosphatase (ALP), osteocalcin (OCN), dentin sialophosphoprotein (DSPP)), and COL1 are expressed in cementoblasts, and hence can be considered as specific markers of cementoblasts differentiation [39, 54]. Moreover, it has been reported that CEMP1 regulates downstream processes toward bone and cementum formation and promotes the proliferation and migration of periodontal ligament cells [39, 54]. Therefore, our study investigated the expression of cementoblast-related genes, including CEMP1, SPON1, COL1, OCN, and OPN, using the qRT-PCR assay in days 7, 14, and 21 after treatment. As shown in Fig. 6, the differentiated samples showed a significant increase in the expression of genes in days 7, 14, and 21 days compared to the control. Moreover, the greatest gene expression was observed in day 21. In accordance with our findings, Kadokura et al. reported that in day 21, the CEMP1 gene was most greatly expressed in human periodontal ligament cells [48].

Previous studies have reported that activation of the Wnt-ßcatenin and Wnt signaling pathway has led to an increase in the expression of COL1, OCN, and OPN [38, 55]. Therefore, in our study, the increased expression of the mentioned genes in day 21 may have been due to the activation of this signaling pathway. Also, SPON1 expression was greater in days 7, 14, and 21 compared to the control group. Thus, the greatest level of gene expression was observed in day 21. In Kitagawa et al. study, the expression of F-Spondin mRNA was observed only in cementoblast but not in osteoblasts or periodontal ligament cells. Moreover, it was observed that the increased expression of F-Spondin caused a change in the morphology of the cells, as well as an increase in the expression of ALP, OCN, and OPN mRNA, and ALP activity in HPL cells, suggesting that F-Spondin may affect the differentiation of periodontal ligament cells. Also, ALP, OCN, and OPN were expressed by cementoblasts and osteoblasts, which can play an essential role in mineralization [16]. In this regard, the increased F-Spondin in our study confirmed the increase of OCN and OPN, which subsequently affect the differentiation of the SCAPs cells into cementoblast cells.

In Kadokura et al. study, the expression level of CEMP1 in human periodontal ligament cells in days 7 and 14 was approximately 1.5 and 2.3 folds greater than in day 5, respectively. This result demonstrated an increase in CEMP1 over time, which may be attributable to an increase in the adaptation of isolated cells to the differential microenvironment. However, the level of CEMP1 expression in day 21 was slightly lower than in day 14. It was determined that the decrease in CEMP1 expression after 14 days was due to cell apoptosis [48]. In day 21, the greatest level of CEMP1 gene expression was observed, which can be attributed to the role of CEMP1 gene in reducing periodontal ligament expression and increasing cementum expression, as the increased expression of CEMP1 gene reduces periodontal ligament expression and increases cementum expression [39].

The differentiation of osteoprogenitors is under the regulation of various signaling pathways, a number of transcriptional factors that fundamentally determine the fate of stem cells toward a “mineralizing-like” phenotype [56, 57]. In this study, CEMP1, and CAP, as well as bone-related genes ALP, OCN, DSPP were significantly upregulated [58]. In our study, osteogenic differentiation medium contains Ascorbic acid. CEMP1 were upregulated by Ascorbic acid treatment which plays a direct role in the differentiation of hSCAPs to a "mineralizing-like" phenotype by activating β-catenin signaling cascade [59]. On the other hand, CEMP1 induces the formation of amorphous calcium phosphate (ACP) sphere and has a significant effect on the hydroxyapatite formation.

In addition, till 21 days of differentiation, the ALP activity were enhanced and the gene expression of OPN, OCN, CEMP1, COL1 and SPON1.

were upregulated. This show the regulatory role of ALP in the proliferation and differentiation of hSCAPs [60]. However, to obtain a close mechanism of hSCAPs differentiation, the Wnt/β-catenin signaling pathway needs to be screened which is in progress in our lab.

## Conclusions

In conclusion, we extracted SCAPs from the root apical of the third human molar and differentiated them into cementoblast cells. Complementary assays of Alizarin red S staining and alkaline phosphatase, as well as qRT-PCR-based analysis of the CEMP1, SPON1, COL1, OCN, and OPN, confirmed the nature of cementoblast cells. The results demonstrated that these cells could be differentiated into cementoblast cells and, may be a viable option for enhancing the osteointegration between the jawbone and the implant. Therefore, the use of cementoblast-derived cells from SCAP could be proposed for periodontal regeneration therapy. Notably, additional in vivo studies are warranted to confirm its application in enhancing osteo-injection and, ultimately, the performance of dental implants.

## Data Availability

The datasets used or analysed during the current study are available from the corresponding author on reasonable request.
