# Correction: Isolation and characterization of apical papilla cells from root end of human third molar and their differentiation into cementoblast cells: an in vitro study

**DOI:** 10.1186/s12575-023-00202-5

**Published:** 2023-05-30

**Authors:** Morvarid Ebadi, Amirfarhang Miresmaeili, Sarah Rajabi, Shahrokh Shojaei, Sareh Farhadi

**Affiliations:** 1 Department of Biomedical Engineering, Central Tehran Branch, Islamic Azad University, Tehran, Iran; 2 Orthodontic Department of Hamadan University of Medical Sciences and Hamadan Dental Research Centre, Hamadan, Iran; 3 Department of Cell Engineering, Cell Science Research Center, Royan Institute for Stem Cell Biology and Technology, ACECR, Tehran, Iran; 4 Stem Cells Research Center, Tissue Engineering and Regenerative Medicine Institute, Central Tehran Branch, Islamic Azad University, Tehran, Iran; 5 Department of Oral & Maxillofacial Pathology, Faculty of Dentistry, Tehran Medical Sciences, Islamic Azad University, Tehran, Iran

**Correction: Biol Proced Online 25, 2 (2023)**

10.1186/s12575-023-00190-6

In the version of this article originally published [1], there was an error in the author's order. The order of authors is now corrected in the author group and the original article has been corrected.
